# Impact of the drip-and-ship model on the treatment of acute ischemic stroke in relation to distance from the thrombectomy center

**DOI:** 10.3389/fneur.2025.1708262

**Published:** 2026-01-12

**Authors:** Franziska Lieschke, Gina Mueller, Fee Keil, Ferdinand O. Bohmann, Christian Grefkes, Jan Hendrik Schaefer

**Affiliations:** 1 Department of Neurology, Goethe University Frankfurt, University Hospital, Frankfurt/Main, Germany; 2 Department of Neurology with Experimental Neurology, Charité Universitätsmedizin, Berlin, Germany; 3 Institute of Neuroradiology, Goethe University Frankfurt, University Hospital, Frankfurt/Main, Germany

**Keywords:** ischemic stroke, endovascular treatment, mechanical thrombectomy, drip-and-ship, direct-to-center, distance

## Abstract

**Background:**

The drip-and-ship model is a common practice for patients with ischemic stroke due to large-vessel occlusion (LVO), providing initial diagnostics and thrombolysis in transition to endovascular treatment (EVT). However, hospital transfer results in treatment delays for patients requiring EVT, potentially affecting outcomes. We sought to explore the association between distance from residence and time intervals to admission with clinical outcomes after EVT.

**Methods:**

In this monocentric retrospective cohort study, patients with acute ischemic stroke due to LVO who underwent EVT at Frankfurt University Hospital between 2017 and 2023 were analyzed. Patients were grouped according to direct-to-center (DC) or drip-and-ship (DS) admission. Clinical outcome parameters included patient global disability after 90 days as measured by the modified Rankin Scale (mRS) and National Institutes of Health Stroke Scale (NIHSS) score improvements analyzed in relation to geographical distance and time metrics. A subgroup analysis based on the distance from residence in 10 km intervals was added.

**Results:**

A total of 334 patients were included. Of these, 41.9% were DC admissions and 58.1% DS were admissions. Distances from home to center were shorter for DC patients (11.1 km vs. 36.4 km, *p* < 0.001), resulting in significantly shorter times from symptom onset to admission (−114 min; 71 min vs. 185 min; *p* < 0.001) and to flow restoration (−88 min; 213 min vs. 301 min; *p* < 0.001). After 90 days, no significant differences in clinical outcomes between DC and DS were observed. However, DC patients living closer than 10 km to the center were more likely to achieve an mRS score <3 (OR 2.995; 95%-CI 1.296–7.318; *p* = 0.012).

**Conclusion:**

Proximity of residence to a thrombectomy center may be advantageous for stroke patients, most likely in association with direct pre-hospital transfers. Distances above 30 km more frequently led to drip-and-ship, which may facilitate care through early diagnostics as, signified by a reduction in the relative time delay to flow restoration.

## Introduction

Endovascular therapy (EVT) has revolutionized acute ischemic stroke treatment and improved patient outcomes. A 2016 meta-analysis of five trials confirmed the effectiveness of EVT over thrombolysis alone, with a number needed to treat (NNT) of 2.6 to improve one modified Rankin Scale (mRS) point, and no difference in symptomatic hemorrhages or mortality (1).

Transporting stroke patients with large-vessel occlusions to EVT-capable centers is crucial; however, geographical distance impacts timely and guideline-compliant care. For example, a 2024 New Zealand study observed long travel times (median >3 h), with half of the eligible patients not receiving EVT but the other half benefiting significantly from EVT (2). A 2023 U.S. study linked shorter transport times to better outcomes (3).

Different models exist: direct-to-thrombectomy centers (DC, frequently also referred to as “mothership”) or initial tissue plasminogen activator (tPA) treatment at a nearby hospital before transfer (“drip-and-ship,” DS). DS expands access but requires coordination and risks inter hospital variability (4). A meta-analysis of 13 studies showed worse 90-day outcomes and higher hemorrhage risk for DS, although mortality and recanalization success were similar. A key finding was that the time from symptom onset to groin puncture was considerably shorter in the DC model than in the DS model (5). However, the largest prospective study on this matter, which was conducted primarily in non-urban areas, found no significant difference between models (6), and a recent retrospective Belgian study reached the same conclusion (7). To adjust for a possible delay in thrombolysis due to DC transport, a North American sensitivity analysis suggested bypassing hospitals for EVT centers within 20 miles (8). Overall, this topic warrants further investigation regarding the scenarios in which the DS paradigm might even facilitate stroke treatment by providing early diagnostics and thrombolysis and when direct transport is beneficial.

Thus, the distance to EVT centers is a critical factor in stroke treatment. Our analysis systematically evaluates patient proximity to large-volume EVT centers in DS/DC admissions, assessing functional outcomes, time to recanalization, and thrombolysis impact.

## Methods

### Study population

We conducted a monocentric retrospective cohort study using prospectively collected data on patients with acute ischemic stroke due to LVO who were admitted to Frankfurt University Hospital, Germany, and underwent EVT between 2017 and 2023. Patients were ≥ 18 years of age, and written consent was obtained from either the patient or a legal representative. The study protocol was approved by the local ethics committee of Goethe University Frankfurt (protocol number 19/16).

Frankfurt University Hospital functions as a comprehensive stroke center within the interdisciplinary neurovascular network Rhine-Main (INVN Rhine-Main), one of the 18 certified neurovascular networks established across Germany in recent years to enhance collaboration and ultimately improve stroke care. The INVN Rhine-Main connects 12 clinics in the Rhine-Main region, linking primary and comprehensive stroke centers. These networks operate through shared standard operating procedures (SOPs), simulation training, and coordinated communication tools such as shared contact lists to enable rapid patient transfers. All centers (including the primary stroke centers) were capable of performing CT/MRI angiography and mismatch imaging.

Patient transport models followed clinical practice in the study region, where the emergency medical service (EMS) lacks standardized pre hospital protocols such as the LAMS, RACE, FAST-ED, or PASS scales for directing suspected large-vessel-occlusion strokes to thrombectomy-capable centers (9–15). As EMS in Germany is organized at the state and municipal levels, practices vary regionally. Based on dispatcher guidance and paramedic judgment, patients with acute neurological symptoms are typically taken to the nearest available hospital having neurology and thrombolysis departments.

To ensure the quality of the retrospectively collected data, patients with insufficient information on home addresses, time of symptom onset, and clinical outcome were excluded from the final analysis. Since the primary objective of this study was to assess the distance between home and hospital, patients with in-hospital strokes or a calculated distance of >100 km, suggesting an event that took place while traveling, were also excluded.

### Clinical variables

Baseline parameters, time metrics (time window from symptom onset to admission, time to initiation of treatment, and time to flow restoration), as well as treatment modalities, were recorded as part of routine clinical care. In cases of failed recanalization, no time to flow restoration was recorded, but patients were included for analyses of clinical outcomes. The linear distance in kilometers between the patients´ home addresses and the University Hospital Frankfurt was calculated based on zip codes with an Excel tool (available under https://userpage.fu-berlin.de/~kweinert/kwluftlinie), which is based on the geonames.org project.^1^ Flow restoration was assessed during EVT (at the end of the procedure). A Thrombolysis In Cerebral Infarction (TICI) score of > 0 generated a standardized procedural timestamp that is routinely documented during thrombectomy, regardless of the eventual success of the intervention. Patients were followed up for 90 days after stroke, at which point a telephone interview was conducted to assess the clinical outcomes.

### Outcome measures

To assess the impact of distance and inter hospital transfer on stroke care, we analyzed and compared geographical distances and time intervals between patients with DC and DS. These metrics were correlated with each other, as well as with the initial stroke severity, as measured by the NIHSS at admission. The primary outcome was global patient disability as measured on the modified Rankin Scale (mRS) after 90 days, which was compared between DC and DS patients, stratified by treatment with or without intravenous thrombolysis. A favorable outcome was defined as an mRS score of < 3. In addition, we examined associations between distance and time metrics with the 90-day mRS and, as a secondary outcome, with NIHSS reduction during the hospital stay, analyzed separately for the DC and DS patient groups. A subgroup analysis based on the distance from residence in 10 km intervals was added, including a binary logistic regression analysis based on the distance from the treating center (<10 km vs. > 10 km).

### Statistical analysis

Data analysis was performed using the Statistical Package for Social Sciences (SPSS, version 29.0.2.0.0, Armonk, NY, United States) and R (R package version 4.3.3). Data were assessed for normal distribution using the Kolmogorov–Smirnov test. The significance of differences in categorical data was calculated using the χ^2^-test. Ordinal and metric data without a normal distribution were assessed using Mann–Whitney *U*-test. We quantified the correlation between geographical distance, time metrics, initial stroke syndrome severity, and outcome parameters using Spearman’s *ρ*. The primary endpoint of mRS after 90 days was analyzed with an ordinal logistic regression of mRS as a common odds ratio (cOR) between direct-to-center and drip-and-ship-admissions, with adjustment for age, sex, NIHSS at admission, pre-stroke mRS, and thrombolysis. The patients were further grouped based on the distance from their homes to the thrombectomy center in 10 km intervals (<10, 10–20, 20–30, 30–40, 40–50, and >50 km). Median mRS scores were compared between these groups. All tests of hypotheses were two-tailed, and a *p*-value of < 0.05 was considered significant.

## Results

### Study population

**Figure 1:**
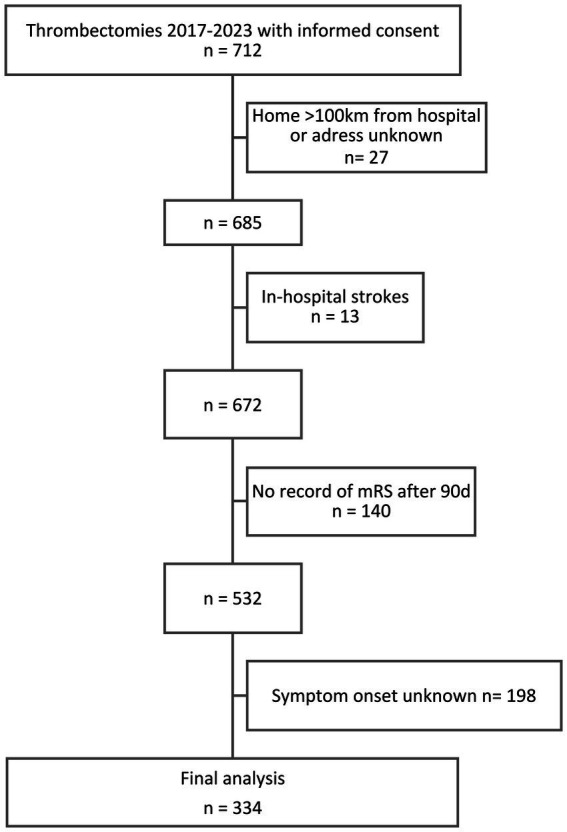
Flowchart of patient exclusion and inclusion criteria. Overall, between 2017 and 2023, *n* = 712 thrombectomies were recorded, of which *n* = 334 were included in the final analysis.

Between January 2017 and December 2023, 712 patients with ischemic stroke were enrolled and underwent EVT at Frankfurt University Hospital. After exclusion for lack of data on addresses, time of symptom onset, and outcome, as well as in-hospital stroke, 334 patients were included in the final analysis (Figure 1).

Direct transfers via emergency medical services accounted for 140 (41.9%) and drip-and-ship admissions for 194 (58.1%) patients. The administration of bridging thrombolysis was similar in both groups, with 87 (62.1%) patients in the DC group and 122 (62.9%) patients in the DS group. Baseline characteristics of all patients grouped by DC and DS are detailed in Table 1. No significant differences were found between the groups for all clinical baseline parameters.

**Table 1:** Baseline characteristics for all patients grouped by direct-to-center (DC) and drip-and-ship (DS) admission modes.

Parameter			All	Direct-to-center (DC)	Drip-and-ship (DS)	Significance (P)
Number			334	140 (41.9%)	194 (58.1%)	
Age (years; mean; standard deviation)			68.2 ± 14.3	69.4 ± 15.2	70.1 ± 13.2	0.89^1^
Female sex (n; %)			159 (48%)	66 (47%)	93 (48%)	0.96^2^
Medical history	Arterial hypertension		258 (77%)	106 (76%)	152 (78%)	0.57^2^
	Diabetes		60 (18%)	28 (20%)	32 (17%)	0.41^2^
	Dyslipidemia		86 (26%)	37 (26%)	49 (25%)	0.48^2^
	Prior stroke		49 (15%)	20 (14%)	29 (15%)	0.87^2^
	Atrial fibrillation	Prior	87 (26%)	35 (25%)	52 (27%)	0.72^2^
		Newly diagnosed	67 (20%)	26 (19%)	41 (21%)	
Oral anticoagulation	Vitamin K antagonists		11 (3%)	5 (4%)	6 (3%)	0.99^2^
	Direct oral anticoagulants		47 (14%)	19 (14%)	28 (14%)	
Occluded vessel	Internal carotid artery		86 (26%)	30 (21%)	56 (29%)	0.23^2^
	Middle cerebral artery		214 (64%)	94 (67%)	120 (62%)	
	Posterior cerebral artery		2 (0.6%)	2 (1%)	0 (0%)	
	Basilar artery		31 (9%)	14 (10%)	17 (9%)	
	Vertebral artery		1 (0.3%)	0 (0%)	1 (0.5%)	
Alberta Stroke Program Early CT Score (ASPECTS; median; IQR)			9 (7–10)	9 (7–10)	9 (7–10)	0.97^1^
mRS prior to stroke (median; IQR)			0 (0–1)	0 (0–1)	0 (0–1)	0.60^1^
NIHSS at admission (median; IQR)			13 (8–17)	13 (7–17)	14 (9–18)	0.11^1^
TICI score (median, IQR)			2b (2b-3)	2b (2b-3)	2b (2b-3)	0.24^1^

1 = Mann–Whitney *U* test, 2 = χ^2^ test.

### Distances and procedural metrics

**Figure 2:**
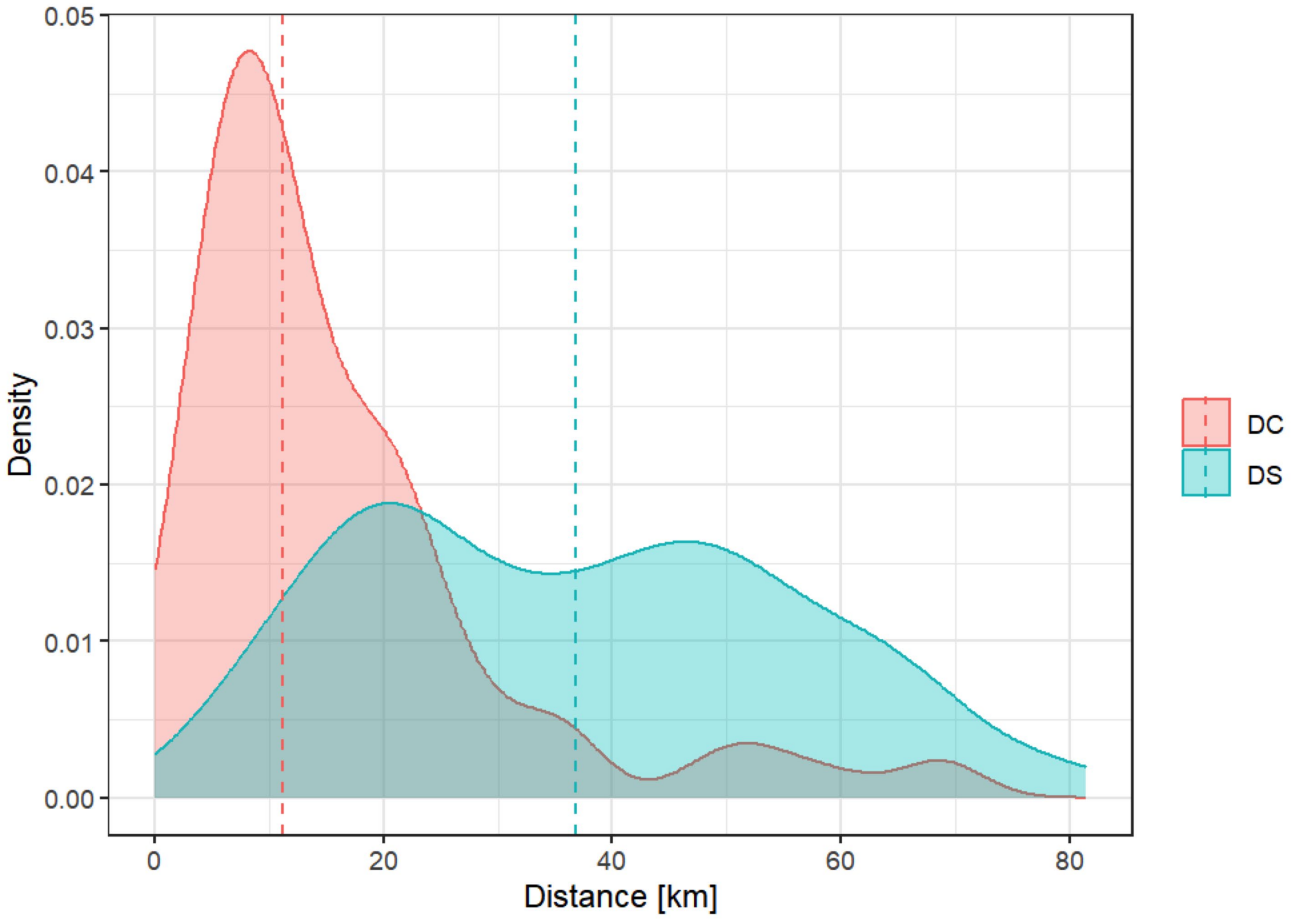
Density plot of the calculated distance between home and thrombectomy center for direct-to-center (DC, red) and drip-and-ship (DS, blue) patients. DC patients were located significantly closer to the hospital than DS patients (median 11.1 km; interquartile range [IQR] 6.7–21.3 km vs. 36.4 km; IQR 20.4–50.8 km; *p* < 0.001).

Differences in spatial distance from home addresses to the thrombectomy center were significantly shorter for DC compared to DS admissions (median 11.1 km; interquartile range [IQR] 6.7–21.3 km vs. 36.4 km; IQR 20.4–50.8 km; *p* < 0.001; Figure 2).

Time from symptom onset to admission was similarly shorter for DC compared to DS (median 71 min; IQR 52–101 min vs. 185 min; IQR 143–246 min; *p* < 0.001). Likewise, the time interval between symptom onset and flow restoration was significantly shorter in the DC group than in the DS group (median 213 min; IQR 160–269 min vs. 301 min; IQR 249–369 min; *p* < 0.001; Table 2). There were significant positive correlations between spatial distance and time from symptom onset to admission for all patients (Spearman *ρ* = 0.431; *p* < 0.001), for patients with DC (ρ = 0.237; *p* = 0.005), and less pronounced for patients with DS (ρ = 0.145; *p* = 0.043; Table 3; Supplementary Figure S1). The NIHSS at admission only showed a positive correlation with distance in DC admissions (ρ = 0.169; *p* = 0.0458), but not in DS (ρ = −0.002; *p* = 0.9769; Table 3).

**Table 2:** Distances between home addresses and thrombectomy center (based on zip codes) and time intervals between symptom onset and admission/successful recanalization for all patients and grouped by direct-to-center (DC) and drip-and-ship (DS) admission modes.

Parameter	All	Direct-to-center (DC)	Drip-and-ship (DS)	Significance (P)
Distance from home to hospital (km; median; IQR)	21.4 (11.1–44.3)	11.1 (6.7–21.3)	36.4 (20.4–50.8)	<0.001^1^
Time from symptom onset to admission (minutes; median; IQR)	140 (73–203)	71 (52–101)	185 (143–246)	<0.001^1^
Time from symptom onset to successful recanalization (minutes; median; IQR)	269 (203–343)	213 (160–269)	301 (249–369)	<0.001^1^

1 = Mann–Whitney *U* test.

**Table 3:** Secondary analyses of correlations between geographical distance, time metrics, initial stroke syndrome, severity and outcome parameters using Spearman’s ρ.

Correlations	All	Direct-to-center (DC)	Drip-and-ship (DS)
Distance ~ Time from symptom onset to admission	ρ = 0.431 *p* < 0.001	ρ = 0.237 *p* = 0.005	ρ = 0.145 *p* = 0.043
Distance ~ Time from symptom onset to flow restoration	ρ = 0.328 *p* < 0.001	ρ = 0.134 *p* = 0.134	ρ = 0.091 *p* = 0.225
Distance ~ NIHSS at admission	ρ = 0.187 *p* < 0.001	ρ = 0.169 *p* = 0.046	ρ = −0.002 *p* = 0.977
Distance ~ NIHSS reduction	ρ = −0.020 *p* = 0.744	ρ = −0.019 *p* = 0.842	ρ = 0.014 *p* = 0.858
Distance ~ mRS after 90 days	ρ = 0.023 *p* = 0.676	ρ = 0.025 *p* = 0.766	ρ = 0.076 *p* = 0.295
Time from symptom onset to admission ~ NIHSS at admission	ρ = −0.133 *p* = 0.015	ρ = −0.248 *p* = 0.003	ρ = −0.201 *p* = 0.005
Time from symptom onset to admission ~ NIHSS reduction	ρ = 0.138 *p* = 0.020	ρ = 0.239 *p* = 0.010	ρ = 0.168 *p* = 0.030
Time from symptom onset to admission ~ mRS after 90 days	ρ = −0.010 *p* = 0.850	ρ = 0.130 *p* = 0.125	ρ = −0.033 *p* = 0.645
Time from symptom onset to flow restoration ~ NIHSS reduction	ρ = 0.286 *p* < 0.001	ρ = 0.426 *p* < 0.001	ρ = 0.284 *p* < 0.001
Time from symptom onset to flow restoration ~ mRS after 90 days	ρ = 0.029 *p* = 0.618	ρ = 0.090 *p* = 0.316	ρ = 0.033 *p* = 0.662

### Clinical outcome

**Figure 3:**
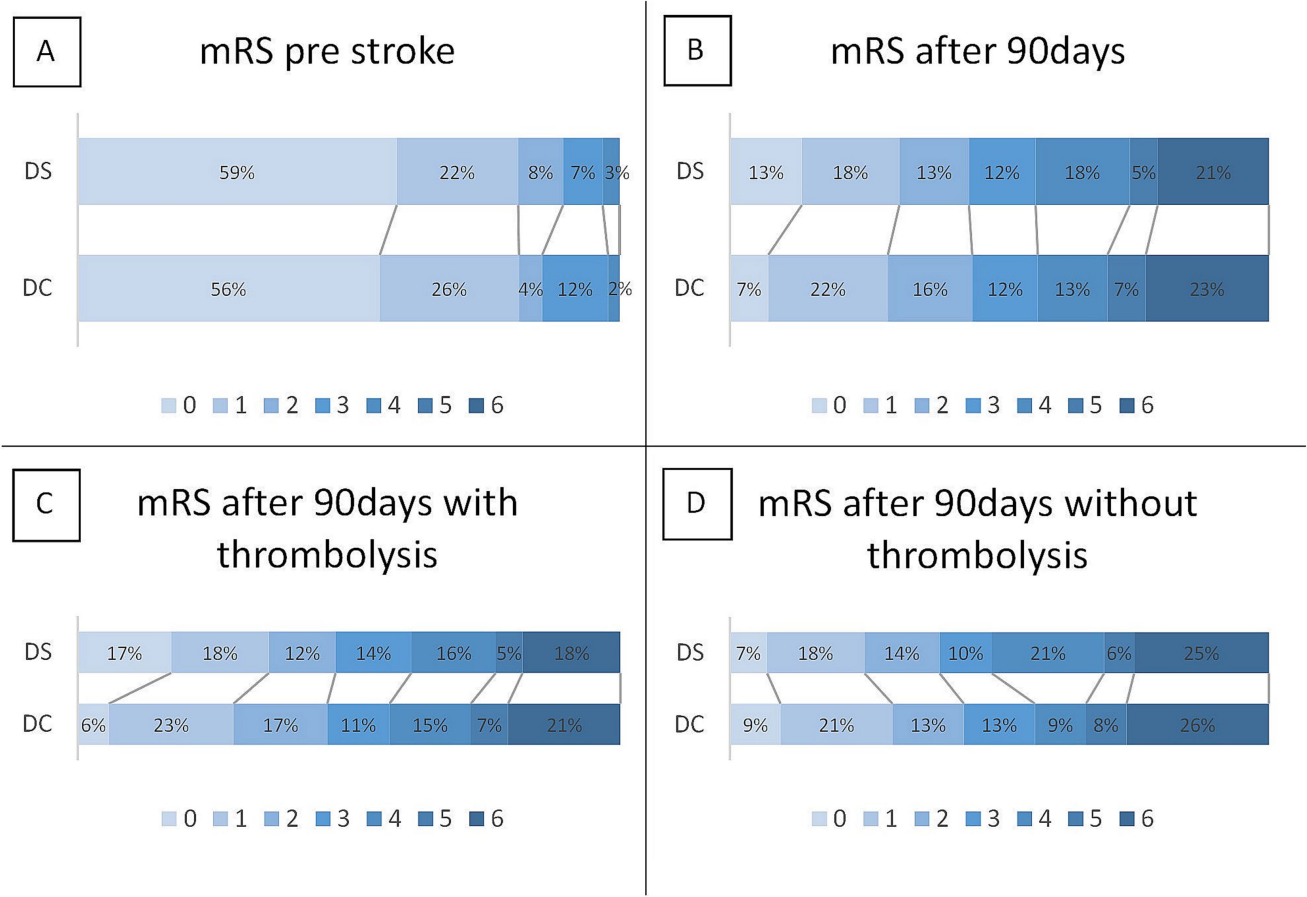
Clinical outcomes based on the modified Rankin Scale (mRS) for direct-to-center (DC) and drip-and-ship (DS) patients. **(A)** mRS score before the stroke event. **(B)** mRS 90 days after stroke obtained by telephone interview and stratified by **(C)** administration of thrombolysis and **(D)** absence of thrombolytic therapy before recanalization. After 90 days of the index stroke, there was no significant difference in the mRS between the DC and DS groups (median 3; IQR 1–5 vs. 3 IQR 1–5; *p* = 0.54). In patients not receiving thrombolysis, there was a shift toward worse outcomes, especially for DS compared to DC patients, albeit this was also not significant (median 4; IQR 1.25–175 vs. median 3; IQR 1–6 vs. *p* = 0.667).

After 90 days of the index stroke, there was no significant difference in mRS between the DC and DS groups (median 3; IQR 1–5 vs. 3 IQR 1–5; *p* = 0.54; Table 4). In the ordinal logistic regression analysis of mRS, with adjustment for age, sex, NIHSS score at admission, pre-stroke mRS, and thrombolysis, there was also no statistical evidence for a lower mRS for DC compared to DS (cOR 0.806; 0.543–1.195; *p* = 0.283). In further analysis, the median mRS was lower for DC compared to DS in cases without the administration of thrombolysis, which was not significant (median, 3; IQR, 1–6 vs. median, 4; IQR, 1.25–5.75; *p* = 0.667; Figure 3).

**Table 4:** Clinical outcomes measured on the modified Rankin scale (mRS) and National Institutes of Health Stroke Scale (NIHSS), as well as hospitalization time for all patients grouped by direct-to-center (DC) and drip-and-ship (DS) admission mode.

Parameter	All	Direct-to-center (DC)	Drip-and-ship (DS)	Significance (P)
mRS at discharge (median; IQR)	3 (1–4)	3 (1–5)	3 (1–5)	0.52^1^
mRS after 90 days (median; IQR)	2 (1–4)	3 (1–5)	3 (1–5)	0.54^1^
NIHSS at discharge (median; IQR)	3 (1–8)	3 (1–7)	4 (1–10)	0.35^1^
NIHSS reduction between admission and discharge (median; IQR)	−7.5 (−13–−3)	−7 (−13–1)	−7 (−12–−3)	0.70^1^
Days in hospital (n; median; IQR)	10 (7–14)	9 (6–12)	10 (6–15)	0.12^1^

mRS after 90 days was obtained by telephone interviews. 1 = Mann–Whitney *U* test.

Overall, no significant correlation was found between the distance from home to the thrombectomy center and mRS after 90 days (all patients *ρ* = 0.023; *p* = 0.676) or time from symptom onset to admission and mRS after 90 days (ρ = −0.010; *p* = 0.850). On the other hand, NIHSS reduction from admission to discharge was significantly correlated with the time from symptom onset to flow restoration (*n* = 261; ρ = 0.286; *p* < 0.001). This observation was stronger in patients with DS (*n* = 106; ρ = 0.426; *p* < 0.001) than in patients with DC (*n* = 155; ρ = 0.284; *p* < 0.001; Table 3).

After stratification for distances from patients´ homes to the thrombectomy center in 10 km steps, no significant between-group differences could be detected for the median mRS, although a stepwise increase was noted in DC patients from <10 km (median 2), 10–20 km (median 3), and 20–30 km (median 4). In the binary logistic regression analysis of mRS after 90 days with adjustment for age, sex, NIHSS at admission, pre-stroke mRS, and thrombolysis, DC patients had an odds ratio of 2.995 (95% CI 1.296–7.318; *p* = 0.012) to achieve a favorable outcome if living < 10 km from the admitting thrombectomy center compared to patients living > 10 km away (Supplementary Figure S2). The study area included both urban regions and sparsely populated (sub)rural areas with limited infrastructure (Supplementary Figure S3).

## Discussion

In this retrospective analysis of the proximity of residence to a large thrombectomy center, greater distances correlated with longer treatment delays from symptom onset to recanalization, but not generally with worse functional outcomes. Overall, reducing admission delays is critical, because early recanalization improves functional outcomes (16, 17). This supports the direct routing of suspected LVO patients to EVT-capable centers, although whether there is merit in the drip-and-ship model is being debated (18–21).

Notably, in our study, the time delay from symptom onset to thrombectomy center admission between DC and DS was greater than the delay from symptom onset to flow restoration by 26 min. This finding is most likely explained by shorter door-to-recanalization times in patients with DS due to preemptive diagnostics in the primary hospital and priming of the thrombectomy center. Despite faster treatment times in the DC group, 90-day functional outcomes (mRS) were similar between the DC and DS groups, aligning with some previous studies (6, 7). Particularly, the randomized RACE-CAT trial failed to support a general direct-to-center strategy (6). A similar finding was derived from the TRIAGE-STROKE study, which was a multicenter randomized trial in a broader region of Denmark (22). As TRIAGE-STROKE was terminated early, it was underpowered to demonstrate a significant difference in functional outcomes on day 90. In these studies, time delays remained a disadvantage with the DS model, which is also true in our study. On the other hand, bypassing the nearest hospital in favor of directly presenting to the thrombectomy-capable centers might cause longer initial transport times. Our study differs in this aspect, as DC patients were living much closer to our center than DS patients. As a result, the median time from symptom onset to hospital admission was shorter in the DC cohort at 71 min. In comparison, the RACECAT trial reported a median onset-to-hospital arrival of 88 min for patients initially presenting to a primary stroke center versus 142 min for DC patients. Similarly, the TRIAGE-STROKE trial reported a median travel time of 81 min to a primary stroke center compared to 177 min for patients with DC. Consistent with these previous studies, patients with DS in the cohort also experienced longer onset-to-flow restoration times, primarily due to additional delays within the primary stroke center and during interhospital transfers.

As expected, DS patients lived farther from the thrombectomy center than DC patients. Distance increased symptom onset-to-admission times, more so for DC than for DS. However, geographic distance does not uniformly translate into transport time, as it is highly dependent on regional infrastructure and road conditions. Our study included both urban and rural areas, where in urban areas, shorter distances may still result in prolonged transport times due to traffic congestion and complex routing. Conversely, in rural settings, although absolute distances are typically greater, emergency services may benefit from direct routes and higher average speeds on highways, resulting in comparable or even shorter transport times per kilometer. In this context, mathematical modeling serves as a critical tool for optimizing stroke care systems. Its ability to simulate various configurations—such as hospital network adjustments, triage algorithms, and transport strategies—enables healthcare planners to evaluate potential impacts on patient outcomes and resource utilization (23). We observed a non-significant, stepwise increase in the mRS for DC patients as distance increased; however, no significant linear correlation was detected. However, patients living within 10 km and admitted directly to a thrombectomy center had better outcomes, suggesting that the strategic placement of these centers is critical to providing optimal care. Bypassing closer hospitals for thrombectomy centers within 20 miles (~ 32 km) may be advisable and aligns with the concentration of DC admissions at this distance in our data (8).

Other strategies, which were not evaluated in our current study, are the use of mobile stroke units (MSU) (24, 25) or decentralized strategically placed CTs (26, 27). Cumulative evidence demonstrated that the use of MSUs not only increased the likelihood of receiving thrombolysis with significantly reduced times from symptom onset to the start of thrombolytic treatment but also correlated with overall better functional outcomes and higher rates of excellent outcomes (28). This benefit extends to EVT through the possibility of avoiding inter hospital transfers (29, 30). As a consequence, the European Stroke Organization endorsed the first international guideline on MSU treatment in 2022 (31). Similarly, strategically placed decentralized CTs, in combination with telestroke-guided diagnosis and thrombolytic treatment by paramedics, resulted in earlier diagnosis and subsequently shorter time to treatment initiation. However, the likelihood of receiving thrombolysis did not increased, which contrasts with the high frequency of thrombolysis in the DS model (26). Both strategies, however, offer the possibility of vessel imaging, allowing for the identification of large-vessel occlusions earlier, thereby impacting faster patient routing to EVT-capable centers with the opportunity of minimizing futile interhospital transfers through the use of telemedicine consultation (32). In the secondary analysis of our data, NIHSS reduction from admission to discharge correlated significantly with the time from symptom onset to flow restoration, especially in DS patients, underscoring the need for high-quality care standards and efficient inter hospital transfers. Previous studies have demonstrated that neurovascular networks enhance stroke treatment through collaborative training and the streamlining of workflows, thereby improving coordination in DS pathways and supporting timely clinical decisions through standardized imaging and treatment protocols. Reducing door-to-needle and door-to-groin times for DS patients remains crucial and can be effectively achieved through joint training initiatives and harmonized stroke management protocols (33–35). In this regard, a pre defined transport strategy from primary stroke centers to a thrombectomy-capable hospital has been shown to effectively reduce delays while maintaining the chance of early thrombolysis in patients with LVO (36).

Additional subgroup analyses showed a higher median mRS score for DS patients who did not receive thrombolysis, although the difference was not statistically significant. Given that previous studies have linked studies linking DS delays to worse outcomes, our lack of significance may stem from a smaller sample size (5, 37, 38). The observed trend toward worse outcomes in non-thrombolysis cases reinforces the need for early identification of thrombolysis eligibility by timely allocation to certified stroke units, where expediting thrombolysis and transport to EVT presents the highest priority. Although both intravenous thrombolysis and EVT are highly effective therapies, their benefits remain time-dependent (1, 39). In this study, no significant correlation was found between the time to recanalization and the 90-day mRS score, likely due to sample size limitations.

Thrombolysis is generally administered as alteplase. Recent evidence suggests that tenecteplase may offer advantages over alteplase in the treatment of LVO strokes, particularly in the DS model. The pharmacologic profile of tenecteplase, allowing for single-bolus administration, simplifies logistics and may facilitate faster door-in-door-out times. Some studies, such as EXTEND-IA TNK (40), have shown higher early recanalization rates with tenecteplase compared to alteplase, potentially improving outcomes prior to thrombectomy. However, direct comparisons of tenecteplase and alteplase in DC vs. DS models remain limited, and further data are needed to determine if one agent confers differential benefits depending on the transport strategy.

### Limitations

As a retrospective analysis, our study is prone to biases in data collection and interpretation. Therefore, a substantial number of patients (378/712; 53.1%) were excluded due to insufficient information on treatment times and clinical outcomes or implausible data (e.g., in-hospital strokes, home distances >100 km, suggesting travel activity). Importantly, the exclusion rate of patients with DC and DS was not significantly different (55.3% vs. 51.3%; *p* = 0.29), and this approach ensured inclusion of only complete datasets in the statistical analysis, thereby enhancing data quality and enabling more reliable conclusions. Furthermore, our findings are based on a single thrombectomy center in a highly coordinated neurovascular network, limiting their generalizability to other healthcare systems and transport networks.

Pre hospital factors, such as dispatch and triage protocols, traffic, transport mode (air transport vs. ground transport), and ambulance response times, were not analyzed but could impact outcomes (41–43).

We used home-to-thrombectomy center distance as a surrogate for accessibility, which may not accurately reflect true travel distances or transport times because it does not fully account for all logistical factors affecting the time to treatment (e.g., road infrastructure or ambulance availability). We primarily aimed to systematically analyze regional differences in patient residences and the surrounding infrastructure, including urban, suburban, and remote rural regions.

Our smaller sample size, in comparison to multicentric studies or systematic reviews, limited secondary outcome analyses, preventing a definitive conclusion on DC vs. DS efficacy. The high proportion of DS (58.1%) stands out in comparison to other studies and is evidence of an effective neurovascular network; however, subgroup comparisons (e.g., thrombolysis vs. non-thrombolysis) lacked statistical power. While adjustments were made for age, sex, NIHSS at admission, pre-stroke mRS, and thrombolysis, other unmeasured confounders (e.g., interhospital disparities between primary stroke centers and variations in stroke treatment protocols) may have influenced the results. The analysis of the relationship between time metrics and outcomes using univariate correlation did not account for the potential impact of variability in pre hospital or inter hospital processes, which may particularly account for DS patients, and was not explicitly addressed in the analysis.

## Conclusion

Proximity to a thrombectomy center appears beneficial, particularly when rapid direct transfer is feasible. Distances above 30 km often led to secondary transfers via the DS model, which may help mitigate long transports by enabling early diagnosis and thrombolysis in eligible patients. Strategic placement of thrombectomy centers is crucial for optimizing stroke care. Further systematic meta-analyses have the potential to generate recommendations for clinical practice. One of the findings of our study is that larger distances can be successfully managed via drip-and-ship transport in well-integrated stroke networks.

## Data Availability

The raw data supporting the conclusions of this article will be made available by the authors, without undue reservation.

## Footnotes

^1^ https://www.geonames.org
