# Supplementary material for: Impact of the drip-and-ship model on the treatment of acute ischemic stroke in relation to distance from the thrombectomy center
Source: Front Neurol. 2026 Jan 12;16:1708262. doi: 10.3389/fneur.2025.1708262 (PMC12832375; doi:10.3389/fneur.2025.1708262)
Supplement: Supplementary file 1 [file Data_Sheet_1.docx]

Supplementary Data


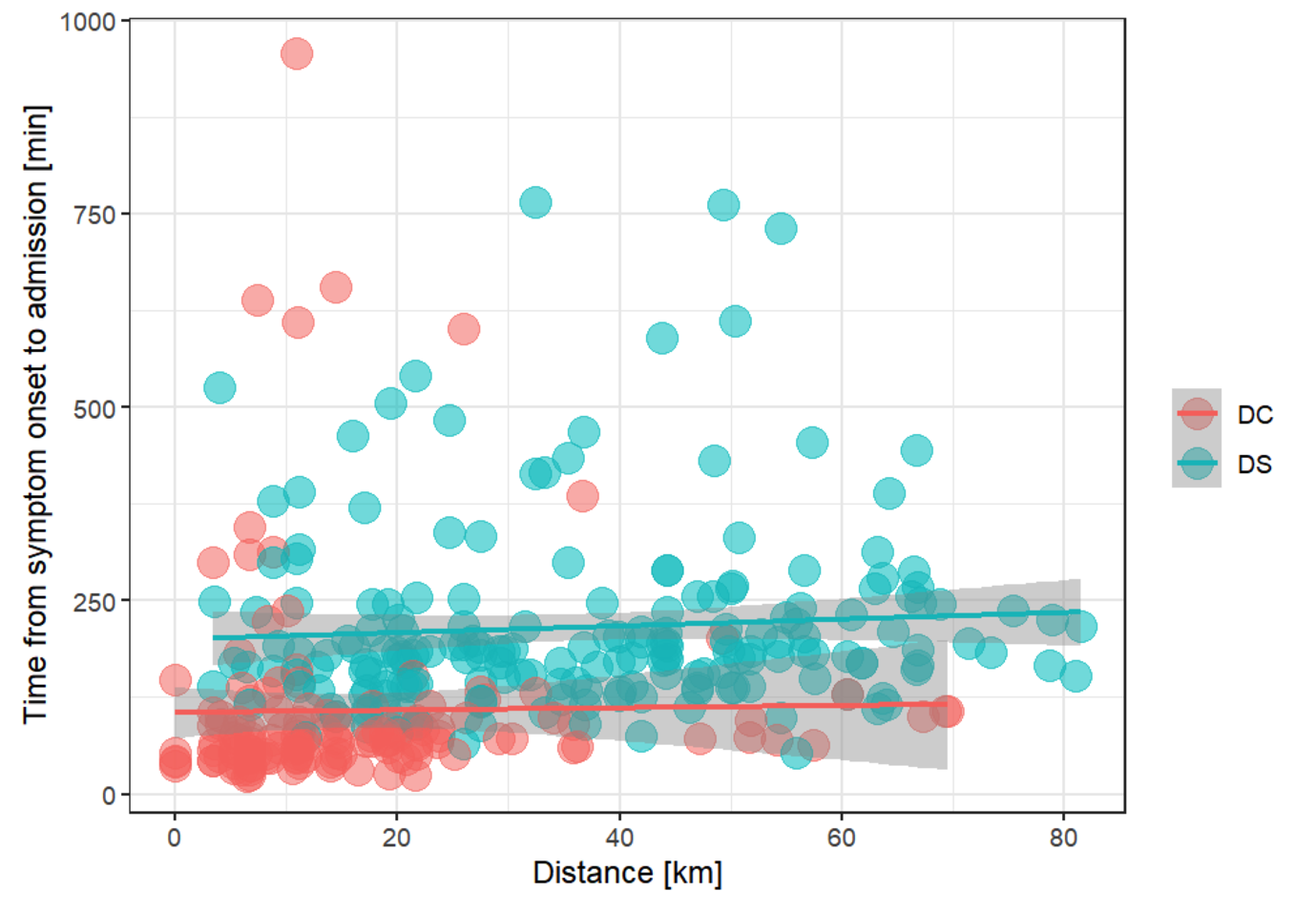


**Supplemental Figure S1:** Correlation between time from symptom onset to hospital admission at the thrombectomy center and distance from patients´ home to the center. Significant positive correlations between spatial distance and the time from symptom onset to admission for all patients (Spearman ρ = 0.431; P <0.001), for DC patients (ρ = 0.237; P = 0.005) and less pronounced for DS patients (ρ = 0.145; p= 0.043) were noted.


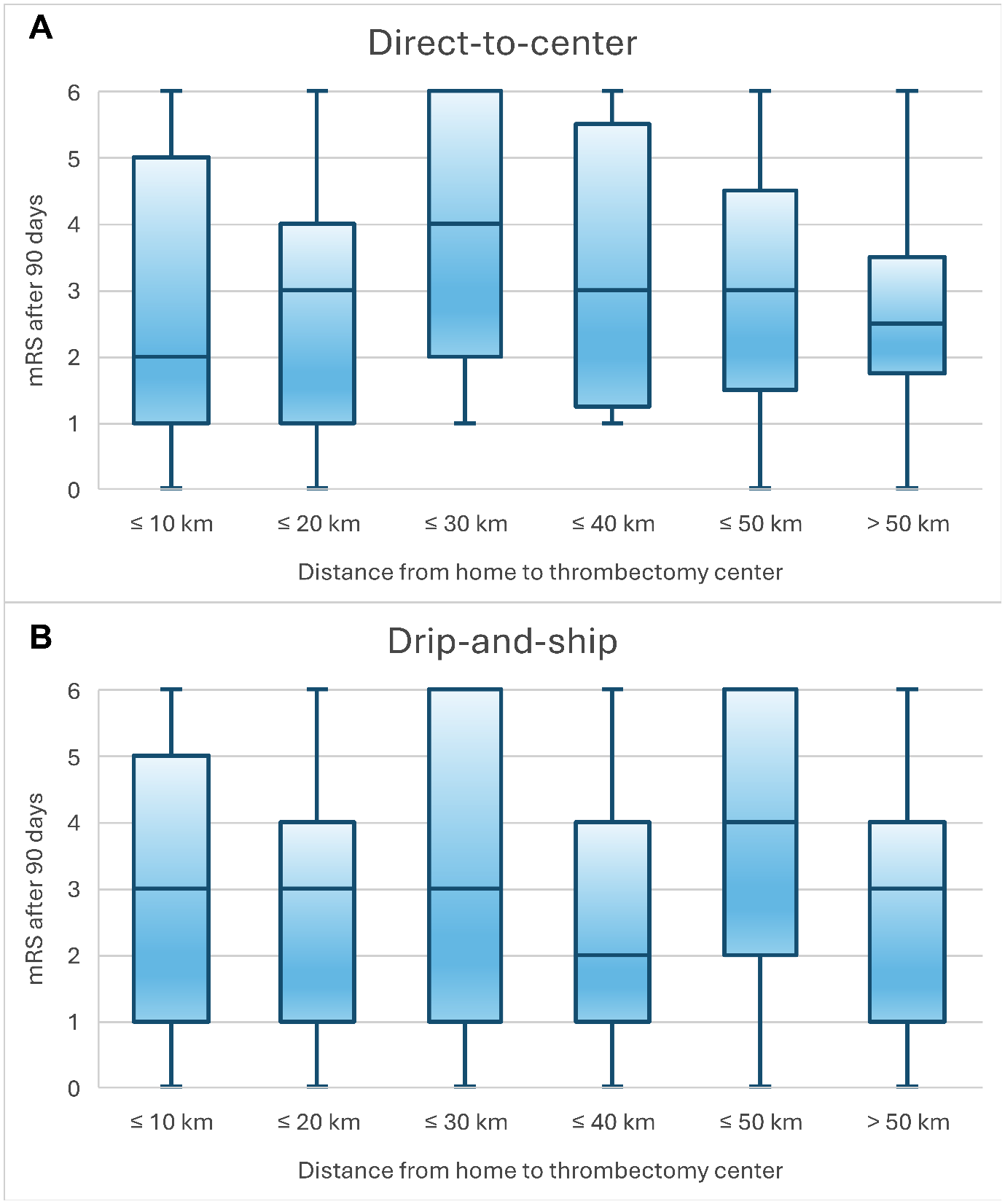


**Supplemental Figure S2:** Box-plot-diagrams of the modified Rankin Scale (mRS) 90 days after the index stroke event A: for direct-to-center (DC) and B: for drip-and-ship (DS) patients with stratification for distance from home to thrombectomy center. No significant differences were detected in the stepwise comparison of distances. In a binary logistic regression analysis of mRS after 90 days with adjustment for age, sex, NIHSS at admission, pre-stroke mRS and thrombolysis, DC patients had an odds ratio of 2.995 (95% CI 1.296 – 7.318; P = 0.012) to achieve a favorable outcome if living < 10km to the admitting thrombectomy center compared to patients living > 10 km away, which was mostly performed as direct-to-center.


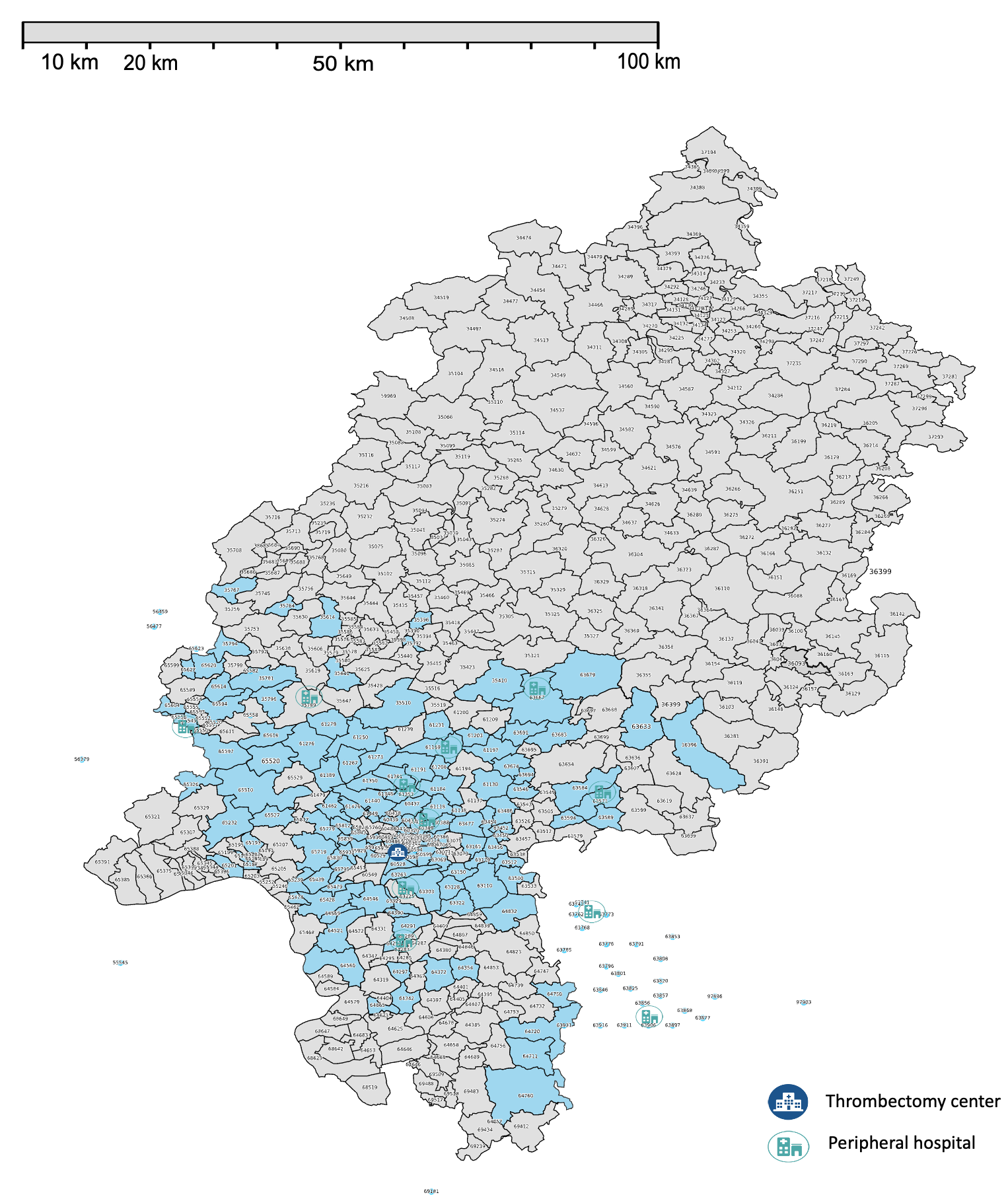


**Supplemental Figure S3:** A scale-accurate map illustrating the state of Hesse, Germany, with the thrombectomy center and peripheral hospitals serving as drip-and-ship hubs. The zip code areas, which align with patient residences are marked in blue. Residences outside of the state are depicted as blue dots with ZIP code.
